# General and age-specific fertility rates in non-affective psychosis: population-based analysis of Scottish women

**DOI:** 10.1007/s00127-022-02313-y

**Published:** 2022-06-01

**Authors:** Angus MacBeth, Paula McSkimming, Sohinee Bhattacharya, John Park, Andrew Gumley, David St Clair, Sarah J. E. Barry

**Affiliations:** 1 University of Edinburgh, Edinburgh, Scotland UK; 2 Robertson Centre for Biostatistics, Institute of Health and Wellbeing, University of Glasgow, Glasgow, Scotland UK; 3 University of Aberdeen, Aberdeen, Scotland UK; 4 NHS Greater Glasgow and Clyde, Glasgow, Scotland UK; 5 Mental Health and Wellbeing, Institute of Health and Wellbeing, University of Glasgow, Glasgow, Scotland UK; 6 Department of Mathematics and Statistics, University of Strathclyde, Glasgow, Scotland UK; 7 School of Health in Social Science, The University of Edinburgh, Rm 2.11, Doorway 6, Medical Quad, Teviot Place, Edinburgh, EH8 9AG Scotland UK

**Keywords:** Schizophrenia, Fertility, Women, Antipsychotics, Psychotic disorders

## Abstract

**Purpose:**

Women diagnosed with non-affective psychosis have a lower general fertility rate (GFR) and age-specific fertility rate (ASFR) than women in the general population. Contemporary data on GFR in this group remain limited, despite substantive changes in prescribing and management. We calculated contemporary estimates of the GFR and ASFR for women diagnosed with non-affective psychosis compared with the general population of women without this diagnosis.

**Methods:**

A population-based design combined routinely collected historical maternity and psychiatric data from two representative areas of Scotland. Women were included from the NHS Grampian or Greater Glasgow and Clyde areas and were aged 15–44 between 2005 and 2013 inclusive. The ‘exposed’ group had a diagnosis of non-affective psychosis (ICD-10 F20–F29) and was compared to the general population of 'unexposed' women in the same geographical areas.

**Results:**

Annual GFR between 2005 and 2013 for women with non-affective psychosis varied from 9.6 to 21.3 live births/1000 women per year in the exposed cohort and 52.7 to 57.8 live births/1000 women per year in the unexposed cohort, a rate ratio (RR) of 0.28 [*p* < 0.001; 95% CI (0.24, 0.32)]. ASFR for all 5-year age groups was lower in the exposed cohort than amongst unexposed women.

**Conclusion:**

We highlight continued low fertility rates in women with a diagnosis of non-affective psychosis, despite widespread availability of prolactin-sparing atypical antipsychotics. Accurate estimation of fertility rates remains crucial in developing needs-matched perinatal care for these women. Methodological improvements using routine datasets to investigate perinatal mental health are also urgently needed.

**Supplementary Information:**

The online version contains supplementary material available at 10.1007/s00127-022-02313-y.

## Introduction

Women with a diagnosis of non-affective psychosis (including schizophrenia) have a lower general fertility rate (GFR), age-specific fertility rate (ASFR), here defined as the presence or absence of offspring, and have lower fecundity (bear fewer children) than women in the general population [1–3]. Importantly, a considerable proportion of women diagnosed with psychotic disorders does bear children [1]. Neonates born to mothers with non-affective psychosis have increased risk of low birth weight, a risk factor for multiple health problems across the lifespan [4]. Furthermore, prescribing of antipsychotics during pregnancy, both typical and atypical, also requires care given that the medication can cross the placental barrier to the developing fetus (5.6). Therefore, mothers with non-affective psychosis and their offspring constitute a high-risk group.

In addition, there is a dearth of evidence around optimal treatment during pregnancy for women with an existing diagnosis of non-affective psychosis, with much of the current guidance being based on general adult treatment options [5, 6]. Emerging evidence suggests that provision of consistent mental health care during pregnancy is associated with lower risk of post-partum psychiatric admission in this group of women [7], and that risk factors for adverse outcomes in pregnancy may be modifiable [8, 9]; however, the health intelligence to support this emerging area remains scarce and under-developed. Establishing reliable population estimates for GFR in this clinical group is a key aspect of the development of perinatal mental health care for women with a diagnosis of non-affective psychosis.

Much of the existing data on fertility rates in pregnancy are based on historical cohorts, with births occurring prior to the introduction of atypical antipsychotics [2]. Contemporary data from a large cohort in Ontario [10, 11] suggest that GFR among women with schizophrenia increased between 1996 and 2009, which the study authors hypothesize may be due to prescription trends in Ontario towards ‘prolactin-sparing’ atypicals, such as Olanzapine, Quetiapine and Ziprasidone, rather than Prolactin-raising typical antipsychotics, or Prolactin-neutral Clozapine. However, data from the UK General Practice records have reported significantly lower fertility rates in women with psychotic disorders compared to normal comparison subjects [1], and data from the UK General Practice records suggested no difference in fertility trends from 1992 to 2007 [12]. Therefore, these data urgently require replication from other datasets and using other linkage methodologies such as birth records.

We took advantage of the availability of high-quality routine datasets in Scotland to generate contemporary estimates of general and age-specific fertility rates in women diagnosed with non-affective psychosis, to provide an evidence base for the fertility rate of this group. Our hypothesis was that GFR and ASFR in the clinical sample would be significantly lower than those for women with no diagnosis of non-affective psychosis.

## Methods

### Design and data sources

We used a population-based design. We combined data from two administrative National Health Service (NHS) health areas in Scotland between the years 2005 and 2013 inclusive:

(i) NHS Grampian (NHSGr) in Northeast Scotland.

1. Aberdeen Maternity and Neonatal Databank (AMND). This dataset holds a record of all obstetric and fertility-related events occurring in women residing in Aberdeen, Scotland, UK since 1950 [13].
2. Psychiatric case records based on Community Mental Health Team contacts for Aberdeen, held in Royal Cornhill Hospital, Aberdeen.

Data were securely transferred to the secure Aberdeen Data Safehaven (DaSH) and transferred by secure VPN to the NHS Greater Glasgow and Clyde (NHSGGC) Safehaven for linkage with the dataset detailed below.

(ii) NHSGGC in West Scotland.

1. Scottish Morbidity Record (SMR)-02 Maternity records. This dataset is held in the NHSGGC Safehaven and records routine data pertaining to obstetric and fertility-related events in the NHSGGC area since 1979.
2. Psychosis Clinical Information System (PsyCIS) for monitoring of all secondary care patients in NHSGGC with a diagnosis of psychosis from secondary care. The PsyCIS register records data for adults (aged 18–65 years) within NHSGGC, in contact with community-based mental health services, presenting with an ICD-10 diagnosis of F20–29, F30–31, F32.3, F33.3 F06.0–06.2, F06.30–06.31 and F1(x) with psychotic symptoms, diagnosed by a consultant psychiatrist using ICD-10 criteria [14]. The dataset covers all incidences of these diagnoses since February 2002.

The PsyCIS dataset was securely transferred to the secure NHSGGC Safehaven.

The maternity and psychiatric datasets were linked and combined in the secure NHSGGC Safe Haven using the Community Health Index (CHI), a unique healthcare identifier allocated to every individual registered with a general practitioner, or born in Scotland. In the absence of CHI, the hospital number was used.

We accessed the combined dataset through the secure VPN connection into the Safe Haven and all outputs were checked for patient identifiability by the NHS Safe Haven team before being released to us. The datasets were completely anonymised and never released outside of the Safe Haven.

Across the four datasets that were ultimately combined, some data had been collected both before 2005 and after 2013. However, this was the period within which the data were considered complete in all of the datasets and so this was defined as the study period.

Favourable ethical opinion was provided by North of Scotland Research Ethics Committee, Caldicott approval by the NHS Grampian Caldicott Guardian, and Local Privacy Advisory Committee (LPAC) approval was obtained for access to the NHS GGC Safehaven.

### Study population

We identified all women aged 15 – 44 at any point between the years 2005 and 2013 inclusive, who had at least one record in the PsyCIS or NHSGr psychiatric data sets with an ICD-10 diagnosis (lifetime or within an episode of care) of non-affective psychotic disorder (F20–F29). These datasets record routine psychiatric data on all patients seen by the secondary community mental health teams for NHS GGC and NHSGr health board areas for an episode of care (inpatient and outpatient) and have been previously used in mental health data science studies [13–16]. We termed this study group the ‘exposed’ group. Women were linked via their CHI to the maternity dataset to identify if they had any births during the years 2005–2013, provided they were in the eligible age range of 15–44 for any relevant year.

As a comparator, we included the general population of women aged 15–44 in NHSGGC and NHSGr at any point between the years 2005 and 2013 inclusive. Again, women were included in the cohort for a particular year if they were in the eligible age range during that year. These data were provided by the National Records of Scotland as aggregate statistics, i.e. the number of women and the number of live births in each of the two areas in each year and by age group. Since the general population would also include the exposed women, we subtracted the number of women and number of live births in our exposed cohort from the general population totals. Therefore, the comparator group could be assumed to include only ‘unexposed’ women, i.e. those with no diagnosis of non-affective psychosis.

### Outcomes

Outcomes were defined as all live births occurring in the study period. Multiple (e.g. twins/triplets) births in the exposed cohort were counted as 1 live birth. We had no information about how multiple births were treated in the unexposed cohort and have assumed that they were treated in the same way as in the exposed group.

### Data Analyses

Annual general fertility rate (GFR) was defined in each group as the total number of live births in that year, multiplied by 1000 and divided by the total number of eligible women in that year (women aged 15 to 44 years). These are presented with corresponding exact binomial 95% confidence intervals (CI) for GFR for each group.

Annual age-specific fertility rates (ASFR) were calculated in 5-year age bands (15–19, 20–24, 25–29, 30–34, 35–39, 40–44 years) for women in the exposed and unexposed cohorts and presented with corresponding 95% CIs for each year, using the same methods as for GFR.

The difference in fertility rates between the exposed and unexposed groups is presented with 95% CIs, calculated using the normal approximation to the binomial distribution. Where there were small numbers in the exposed group for the age-specific rates, the CIs for the difference were not calculated.

The GFRs and ASFRs are presented graphically with 95% CIs for each group, which were smoothed using natural splines for display.

Binomial regression was used to estimate odds ratios comparing fertility rates between study groups and produce corresponding 95% confidence intervals.

## Results

Between 2005 and 2013, there were a total of 212 live births in the exposed cohort, with between 11 and 33 each year. In the unexposed cohort, there were 178,094 live births, with numbers varying by year from around 18,700 to 20,500.

### General fertility rate (GFR)

**Fig. 1:**
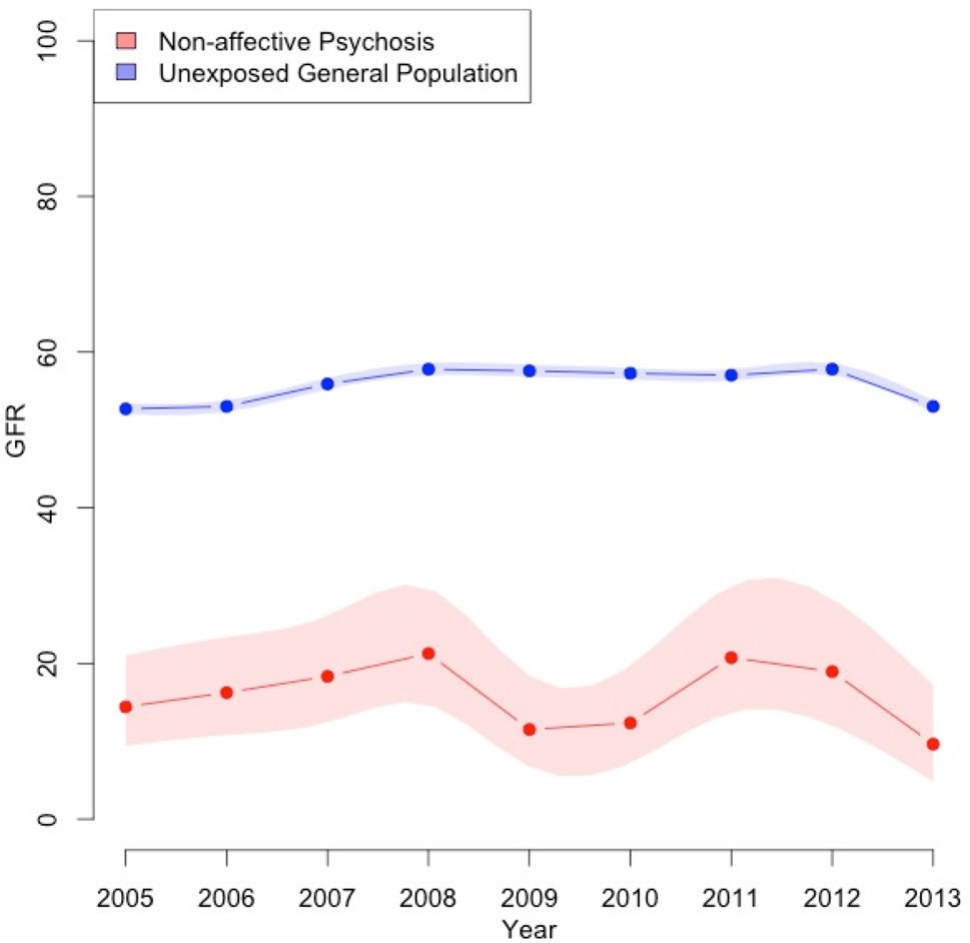
General fertility rate (GFR) per 1000 women with smoothed 95% confidence bands for women aged 15–44 with a diagnosis of non-affective psychosis (exposed) vs women in the general population (unexposed) between 2005 and 2013

Table 1 shows that the GFR in the exposed group varied substantially over the study period, between a high of 21.3 live births/1000 women (95% CI 14.7–29.7) in 2008 to a low of 9.6 live births per 1000 women (95% CI 4.8–17.2) in 2013. Figure 1 demonstrates that there was no systematic drop over the period, rather the GFR continually fluctuated, with various peaks and troughs.

**Table 1:** General fertility rates (GFR) per 1000 women in those aged 15–44 with a diagnosis of non-affective psychosis (exposed), compared with rates amongst women in the general population (unexposed) from 2005 to 2013

Year	Exposed women				General population comparisons				Rate difference	
	Number of births	Number of women	GFR	95% CI	Number of births	Number of women	GFR	95% CI	Difference	95%CI
2005	26	1804	14.4	(9.4, 21.0)	18,688	354,586	52.7	(52.0, 53.4)	38.3	(32.5, 44.1)
2006	28	1724	16.2	(10.8, 23.4)	18,733	353,374	53.0	(52.3, 53.8)	36.7	(30.5, 43.1)
2007	30	1637	18.3	(12.4, 26.1)	19,828	354,669	55.9	(55.2, 56.7)	37.6	(30.7, 44.4)
2008	33	1552	21.3	(14.7, 29.7)	20,511	354,906	57.8	(57.0, 58.6)	36.5	(29.0, 44.1)
2009	17	1478	11.5	(6.7, 18.4)	20,431	354,823	57.6	(56.8, 58.4)	46.1	(40.2, 51.9)
2010	17	1377	12.4	(7.2, 19.7)	20,343	355,229	57.3	(56.5, 58.0)	44.9	(38.7, 51.2)
2011	27	1302	20.7	(13.7, 30.0)	20,316	356,250	57.0	(56.3, 57.8)	36.3	(28.1, 44.5)
2012	23	1214	19.0	(12.0, 28.3)	20,519	354,979	57.8	(57.0, 58.6)	38.9	(30.7, 47.0)
2013	11	1143	9.6	(4.8, 17.2)	18,725	353,187	53.0	(52.3, 53.8)	43.4	(37.2, 49.5)

The unexposed group fluctuated slightly less, which was perhaps a facet of being a much larger population. There was a rise from an early low in 2005 of 52.7 live births/1000 women (95% CI 52.0–53.4) to a high of 57.8 live births/1000 women (95% CI 57.0–58.6) in 2008, after which the GFR stayed reasonably stable until a similar drop to the exposed group in 2013.

The difference in GFR between the groups varied between about 36% to around 46%, but again did not show any particular pattern; rather, it fluctuated across the period and tended to be high when the GFR in the exposed group was particularly low such as in 2013.

### Age-specific fertility rate (ASFR)

**Fig. 2:**
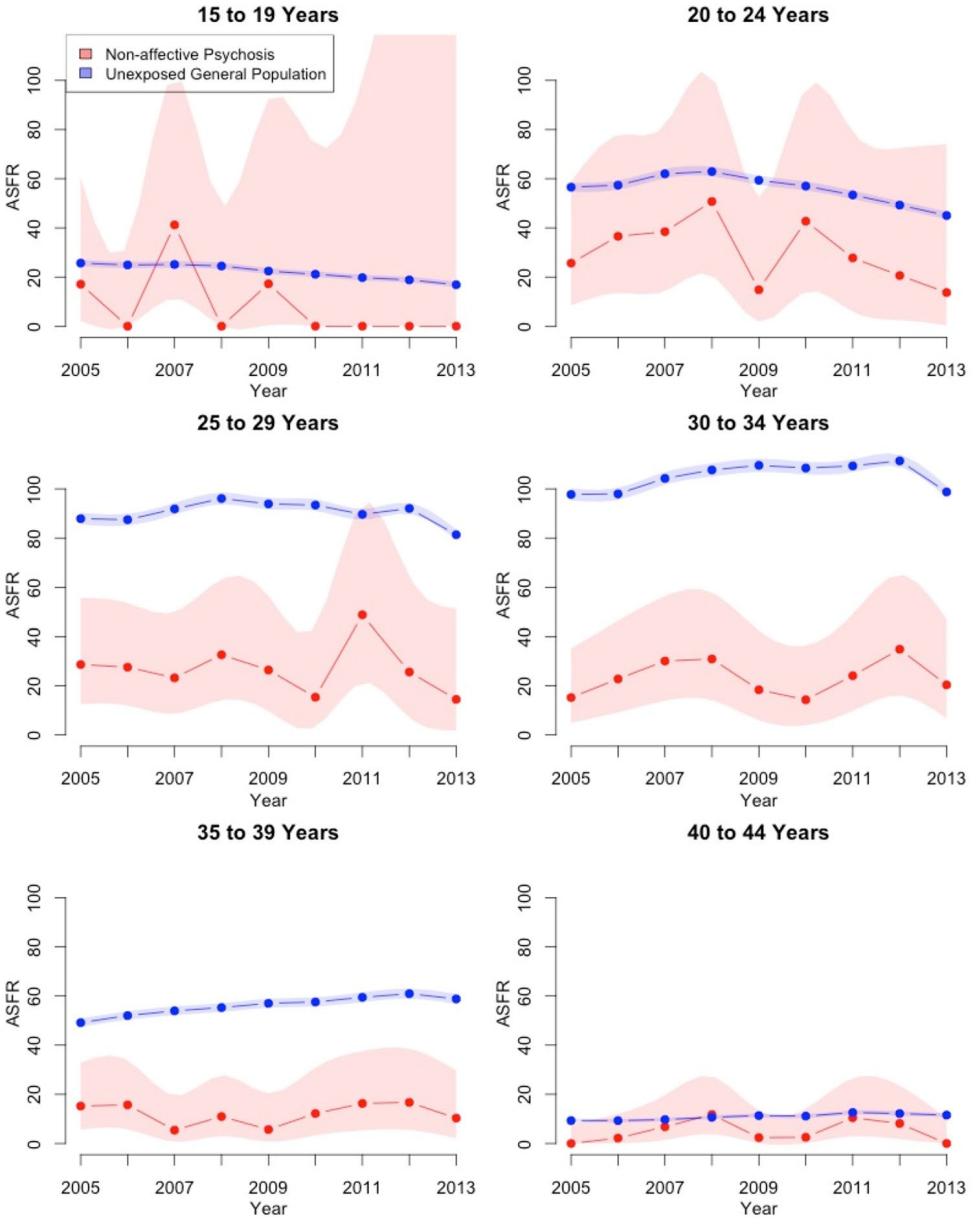
Age-specific fertility rates (ASFR) per 1000 women with smoothed 95% confidence bands by age category for women aged 15–44 with a diagnosis of non-affective psychosis vs women in the general population (unexposed) between 2005 and 2013

The age-specific fertility rates between 2005 and 2013 for age groups 15–19 up to 40–44 years are displayed in Fig. 2 and Supplementary Table 1, with corresponding 95% confidence intervals.

There is a large amount of fluctuation for the youngest and oldest exposed women over the study period, due to very small numbers of births in these groups, making it difficult to make meaningful comparisons to the unexposed women. Consequently, we urge caution in interpreting these data.

Amongst the 20–24-year-olds, there appeared to be a slight drop in the ASFRs over time in both the unexposed and exposed groups; while the 25–34-year-olds were more stable. There appeared an increase in the rate over the study period for unexposed 35–39-year-olds, but this was not apparent for the exposed group, which seemed to be rather more stable.

The 20–29-year-old women tended to have the highest ASFRs amongst the exposed women, while in the unexposed group, the highest rates were amongst 30–34-year-olds, suggesting that the exposed women tended to have babies earlier if they had them.

### Modelling

Results of the regression modelling presented in Table 2 indicate that the exposed group had, on average, a GFR that was around a third of that of the general population [Odds Ratio (OR) = 0.28; 95% CI 0.24–0.32].

**Table 2:** Rate ratio (95% CI) for general (GFR) and age-specific fertility rates (ASFR) per 1000 women comparing those aged 15–44 with non-affective psychosis (exposed) to women in the general population (unexposed)

	Number of births/number of women		
	Exposed women	General population	Group difference rate ratio (95% CI)
Overall GFR	212/13087	178,014/3,192,147	0.28 (0.24, 0.32)
ASFRs			
15 to 19 Years	< 10/570*	10,656/479,274	0.51 (0.24, 1.08)
20 to 24 Years	37/1183	31,603/567,537	0.53 (0.39, 0.74)
25 to 29 Years	53/1952	48,087/531,871	0.28 (0.21, 0.38)
30 to 34 Years	60/2569	51,752/493,055	0.20 (0.16, 0.26)
35 to 39 Years	37/3087	29,510/529,167	0.21 (0.15, 0.28),
40 to 44 Years	18/3726	6406/591,223	0.45 (0.28, 0.71)

*Denotes exact value censored due to small cell count

There was a trend in GFR over time that did not differ between the exposed and unexposed groups, whereby the estimated GFR tended to increase slightly from 2005 to the middle of the study period, before reducing again towards 2013 (Supplementary Fig. 1). There was no evidence of a systematic increase or decrease over the 9 years.

Table 2 shows that the fertility rate was lower for the exposed groups at most ages and.

that the most extreme ratio between the ASFRs of the exposed and unexposed groups was amongst women aged 30–39, with the exposed women having fertility rates of a fifth to a quarter of their unexposed contemporaries. While the fertility rate amongst these unexposed women was higher than for other age groups, this was not the case for the exposed women, whose rates did not vary as much across age groups.

Furthermore, as mentioned previously, the exposed women tended to have higher fertility rates at younger ages than unexposed women, which also contributed to more extreme ratios between the ASFRs amongst the slightly older age groups. Supplementary Fig. 2 displays the estimated trends over time for each age group.

## Discussion

Our data confirm, using contemporary routine datasets, that despite advances in perinatal mental health care, women with a diagnosis of non-affective psychotic disorder have substantially lower general fertility rates than the women without such a diagnosis [1], and that this has changed little over the 9 years of 2005 to 2013 inclusive. Our data suggest a similar pattern within different age groups, with ASFR being significantly lower amongst the exposed than the unexposed women in all age groups between 20 and 39 years.

Our analyses showed no evidence of a systematic change in general fertility rates overall between 2005 and 2013, nor of any difference between the exposed and general population groups in the trend over time, although this may have been affected by the relatively small sample size in the exposed group.

Therefore, on the basis of our data, we did not find substantial evidence that the ubiquity of second generation antipsychotics has led to an improvement in reproductive health in this group [10]. Indeed, our results are more consistent with historical family-based longitudinal data such as the Uppsala Swedish cohort [17], which showed no significant change in fertility rates over time in women with schizophrenia diagnoses over a 40-year timespan in the mid-twentieth century, and with recent UK primary care data that also did not show an increase in reproductive health for this group [12].

A strength of this study is that, alongside the recent UK primary care findings [12], our data are currently the only contemporary estimates of GFR for women with non-affective psychosis in Europe. Our administrative dataset is based on a large sample drawn from a general population and including both urban and rural settings. Therefore, these findings are broadly representative at population level. In this context, the inconsistency of our results with the Ontario findings is worth considering further, given the renewed focus on perinatal care for women with complex psychiatric disorders [6]. As with the other UK study, our data were identified from a mix of public healthcare routine records—in our case secondary care community National Health Service services (for mental health status), maternity hospital data (AMND) and maternity care data (National Records Scotland). This introduces methodological and healthcare system contrasts with the Ontario data, which were derived from hospital admission and public insurance data. Both our data and Hope et al.’s [12] study identified mental health status via entries in clinical records, consistent with ICD-10 diagnoses used in clinical practice [14], whereas the Ontario data directly used ICD diagnostic codes. Therefore, although broadly similar, these differing health system approaches may introduce subtle differences in the application of diagnostic nosology, which could be amplified given the relatively small numbers of women in our dataset identified with non-affective psychosis. This is not to say that one approach to ascertaining case status is superior to the other, but instead to highlight the importance of the health system context in the interpretation of findings across studies. The different fertility rate patterns observed between Ontario and Scotland may also reflect differences in the availability of specialized perinatal care for women with non-affective psychosis (and indeed complex psychiatric disorders in general), differences in prescribing trends [11] and the availability of early intervention for psychosis, which in Scotland was variable during the time period reported on here. Where the findings are consistent across settings is that the fertility rate for women with psychosis continues to be significantly lower than the general population, which emphasizes the need for further work to address the needs of this high-risk population.

A limitation of our study is that bias may have been introduced via the methodology by which the dataset was assembled. For instance, the numbers of women with non-affective psychosis in the dataset reduced across the study period, and the data are relatively small in size, albeit in the context of limited contemporary research in this area. Given that numbers are small for all groups our results should be interpreted with caution. Further, our results are reliant on the quality of routine data, which are subject to variance in recording and sensitive to changes in practice [18]. Using a lifespan diagnosis of psychotic disorder means that puerperal psychoses and women who developed schizophrenia after their pregnancy or had a first psychotic episode during pregnancy were included in the dataset. The level of specificity within the data also precluded us from analyzing sub-patterns of diagnostics, although we also note that the small numbers in the psychosis group would also have introduced issues of statistical power for any such analyses and ethical concerns around confidentiality. These points notwithstanding, we would argue that our pregnancy data are robust, having been derived from routine data sets that have been used in numerous other studies of obstetrics [19]. Further, these data reflect a population cohort from two broad and representative areas of Scotland, combining urban and rural data, with relatively stable population characteristics [13–16, 20].

In addition, we recognize that the identification of psychiatric status was taken from secondary care data; thus, we were unable to capture data on women with a diagnosis of non-affective psychosis whose care is exclusively within primary care. However, given the current Scottish treatment and medication monitoring guidelines for non-affective psychosis, this is likely to account for a very small percentage of cases in the geographical areas under investigation [21]. We also note that our unexposed sample size was achieved by subtracting the psychosis group from the overall sample. It could be argued that, as there is no check on the diagnoses of the unexposed group, there could be double-counting of psychosis. However, since both unexposed and exposed data came from the same sources as our aggregate numbers, there is no reason to believe that the general population minus the count with psychosis is not representative of the number without psychosis. We also note also that there may be some local differences in service provision for treatment of first-episode psychosis (but not subsequent episodes) in this dataset, with early intervention implementation varying across sites in Scotland. However, based on the time period surveyed, this is unlikely to be a significant source of bias in our findings, as the PsyCIS data were drawn from an area with early intervention services throughout the period surveyed, and the NHSGr data from an area without early intervention during the study period. Similarly, the presence of other psychiatric disorders was not an exclusion criterion in the non-psychosis comparator population. Finally, we note that our analyses do not comment on fecundity within our sample, although we recognize that evidence to date suggests that this would also be reduced compared to the general population [3].

Our results highlight several implications for care of women with non-affective psychosis. First, we identify an urgent need to improve understanding of these women’s engagement with and availability of pre-conception planning. In addition to biological explanations of disease transmission, our findings of lower overall fertility lend potential support to a biopsychosocial risk model incorporating social environmental findings that women with a history of schizophrenia have higher rates of adverse experiences [22] and report higher rates of ‘unwantedness’ for pregnancies [23]. Given that GFR is based on live births, terminations and miscarriages would not have been incorporated into the dataset. Previous work has found elevated rates of termination in women with a diagnosis of non-affective psychosis [24, 25] and evidence for an association between abortion and experience of mental health difficulties in general [26]. More work is required to unpick the modifiable risk factors underlying low GFR in this group.

Second, our data support a focus on the 20–35-year age group, not just with regard to the maternity needs of this group, but with regard to general mental health, suggesting potential benefits from the integration of maternity and mental health care. This supports recommendations around early intervention for psychosis [27], and identifies the need to ensure physical health needs are met [28] in conjunction with maternity and mental health care needs.

Third, the risk profile for these women and their children highlights that perinatal mental health pathways need to be further developed to ensure that these women receive best practice, evidence-based care [6], similar to the recognized issues in meeting physical health needs of individuals with psychosis diagnoses [28]. Our psychosis birth data reflect hospital-based deliveries, although the comparator population will also have included home births. This is a discrepancy, but can be contextualized by the extremely low rates of home births in Scotland. Therefore, our data perhaps speak also to a broader needs of all women in relation to maternity care [26]. We also note that the continued evidence of reduced fertility requires greater consideration of the mechanisms by which this reduction persists. In this regard, the current study echoes previous work [1] in highlighting the need for further investigation of socioeconomic factors, health care access and provision of contraceptive and family planning support for this vulnerable group.

Finally, our data further emphasise that improving quality and specificity of routine data recording in both maternity and mental health has the potential to drive forward improvements in delivery of needs-matched care [18].

## Supplementary Information

Below is the link to the electronic supplementary material.

Supplementary Table 1 Age-specific fertility rates (ASFR) per 1000 women amongst those aged 15–44 with a diagnosis of non-affective psychosis (exposed), compared with rates amongst women in the general population (unexposed) from 2005 to 2013. Supplementary Fig. 1 Estimated general fertility rates (GFR) per 1000 women for the population of exposed (solid line) and unexposed (dashed line) women between 2005 and 2013 Supplementary Fig. 2 Estimated age-specific fertility rates (ASFR) per 1000 women for the population of exposed (solid line) and unexposed (dashed line) women between 2005 and 2013 (DOCX 146 KB)
