# Supplementary material for: General and age-specific fertility rates in non-affective psychosis: population-based analysis of Scottish women
Source: Soc Psychiatry Psychiatr Epidemiol. 2022 Jun 1;58(1):105–12. doi: 10.1007/s00127-022-02313-y (PMC9845143; doi:10.1007/s00127-022-02313-y)

Supplementary Table 1

|  |  | **Exposed women** | | | | **Unexposed women** | | | | **Difference** | |
| --- | --- | --- | --- | --- | --- | --- | --- | --- | --- | --- | --- |
| **Age category** | **Year** | **Number of births** | **Number of women** | **ASFR** | **95% CI** | **Number of births** | **Number of women** | **ASFR** | **95% CI** | **Rate difference** | **95% CI** |
| 15 to 19 Years | 2005 | <5 | 117 | 17.1 | ( 2.1,60.4) | 1379 | 53713 | 25.7 | (24.4,27.0) | 8.6 | - |
| 15 to 19 Years | 2006 | 0 | 108 | 0 | ( 0.0,33.6) | 1347 | 54058 | 24.9 | (23.6,26.3) | 24.9 | - |
| 15 to 19 Years | 2007 | <5 | 97 | 41.2 | ( 11.3,102.2) | 1380 | 54923 | 25.1 | (23.8,26.5) | -16.1 | - |
| 15 to 19 Years | 2008 | 0 | 73 | 0 | ( 0.0,49.3) | 1343 | 54809 | 24.5 | (23.2,25.8) | 24.5 | - |
| 15 to 19 Years | 2009 | <5 | 58 | 17.2 | ( 0.4,92.4) | 1231 | 54884 | 22.4 | (21.2,23.7) | 5.2 | - |
| 15 to 19 Years | 2010 | 0 | 48 | 0 | ( 0.0,74.0) | 1151 | 54412 | 21.1 | (20.0,22.4) | 21.1 | - |
| 15 to 19 Years | 2011 | 0 | 35 | 0 | ( 0.0,100.0) | 1047 | 53019 | 19.8 | (18.6,21.0) | 19.8 | - |
| 15 to 19 Years | 2012 | 0 | 19 | 0 | ( 0.0,176.5) | 953 | 50585 | 18.8 | (17.7,20.1) | 18.8 | - |
| 15 to 19 Years | 2013 | 0 | 15 | 0 | ( 0.0,218.0) | 825 | 48891 | 16.9 | (15.8,18.1) | 16.9 | - |
|  |  |  |  |  |  |  |  |  |  |  |  |
| 20 to 24 Years | 2005 | <5 | 195 | 25.6 | ( 8.4,58.8) | 3376 | 59683 | 56.6 | (54.7,58.4) | 30.9 | ( 6.1,55.8) |
| 20 to 24 Years | 2006 | 6 | 164 | 36.6 | (13.5,77.9) | 3440 | 59951 | 57.4 | (55.5,59.3) | 20.8 | (-11.1, 52.6) |
| 20 to 24 Years | 2007 | 6 | 156 | 38.5 | (14.2,81.8) | 3756 | 60554 | 62 | (60.1,64.0) | 23.6 | (-9.9,57.0) |
| 20 to 24 Years | 2008 | 7 | 138 | 50.7 | ( 20.6,101.7) | 3860 | 61337 | 62.9 | (61.0,64.9) | 12.2 | (-28.1, 52.5) |
| 20 to 24 Years | 2009 | <5 | 135 | 14.8 | ( 1.8,52.5) | 3686 | 62072 | 59.4 | (57.5,61.3) | 44.6 | - |
| 20 to 24 Years | 2010 | <5 | 117 | 42.7 | (14.0,96.9) | 3613 | 63366 | 57 | (55.2,58.9) | 14.3 | (-26.7, 55.3) |
| 20 to 24 Years | 2011 | <5 | 108 | 27.8 | ( 5.8,79.0) | 3511 | 65715 | 53.4 | (51.7,55.2) | 25.6 | - |
| 20 to 24 Years | 2012 | <5 | 97 | 20.6 | ( 2.5,72.5) | 3331 | 67586 | 49.3 | (47.7,50.9) | 28.7 | - |
| 20 to 24 Years | 2013 | <5 | 73 | 13.7 | ( 0.3,74.0) | 3030 | 67273 | 45 | (43.5,46.6) | 31.3 | - |
|  |  |  |  |  |  |  |  |  |  |  |  |
| 25 to 29 Years | 2005 | 8 | 279 | 28.7 | (12.5,55.7) | 4581 | 52119 | 87.9 | (85.5,90.4) | 59.2 | (37.7,80.8) |
| 25 to 29 Years | 2006 | 8 | 290 | 27.6 | (12.0,53.6) | 4705 | 53795 | 87.5 | (85.1,89.9) | 59.9 | (39.1,80.6) |
| 25 to 29 Years | 2007 | 6 | 258 | 23.3 | ( 8.6,49.9) | 5199 | 56602 | 91.8 | (89.5,94.3) | 68.6 | (48.1,89.1) |
| 25 to 29 Years | 2008 | 8 | 245 | 32.6 | (14.2,63.3) | 5667 | 58976 | 96.1 | (93.7,98.5) | 63.4 | (39.0,87.9) |
| 25 to 29 Years | 2009 | 6 | 227 | 26.4 | ( 9.8,56.6) | 5668 | 60363 | 93.9 | (91.6,96.3) | 67.5 | (44.3,90.7) |
| 25 to 29 Years | 2010 | <5 | 195 | 15.4 | ( 3.2,44.3) | 5721 | 61241 | 93.4 | (91.1,95.8) | 78 | - |
| 25 to 29 Years | 2011 | 8 | 164 | 48.8 | (21.3,93.9) | 5572 | 62159 | 89.6 | (87.4,91.9) | 40.9 | ( 4.8,77.0) |
| 25 to 29 Years | 2012 | <5 | 156 | 25.6 | ( 7.0,64.3) | 5781 | 62801 | 92 | (89.8,94.3) | 66.4 | - |
| 25 to 29 Years | 2013 | <5 | 138 | 14.5 | ( 1.8,51.4) | 5193 | 63815 | 81.4 | (79.3,83.5) | 66.9 | - |
|  |  |  |  |  |  |  |  |  |  |  |  |
| 30 to 34 Years | 2005 | <5 | 328 | 15.2 | ( 5.0,35.2) | 5509 | 56369 | 97.7 | ( 95.3,100.2) | 82.5 | (67.5,97.5) |
| 30 to 34 Years | 2006 | 7 | 307 | 22.8 | ( 9.2,46.4) | 5261 | 53703 | 98 | ( 95.5,100.5) | 75.2 | (56.6,93.7) |
| 30 to 34 Years | 2007 | 9 | 299 | 30.1 | (13.9,56.4) | 5426 | 52038 | 104.3 | (101.7,106.9) | 74.2 | (52.9,95.4) |
| 30 to 34 Years | 2008 | 9 | 291 | 30.9 | (14.2,57.9) | 5541 | 51453 | 107.7 | (105.0,110.4) | 76.8 | (55.0,98.6) |
| 30 to 34 Years | 2009 | <5 | 272 | 18.4 | ( 6.0,42.4) | 5733 | 52312 | 109.6 | (106.9,112.3) | 91.2 | ( 73.2,109.2) |
| 30 to 34 Years | 2010 | <5 | 279 | 14.3 | ( 3.9,36.3) | 5844 | 53873 | 108.5 | (105.9,111.1) | 94.1 | - |
| 30 to 34 Years | 2011 | 7 | 290 | 24.1 | ( 9.8,49.1) | 6094 | 55725 | 109.4 | (106.8,112.0) | 85.2 | ( 65.6,104.8) |
| 30 to 34 Years | 2012 | 9 | 258 | 34.9 | (16.1,65.2) | 6444 | 57841 | 111.4 | (108.9,114.0) | 76.5 | ( 52.0,101.0) |
| 30 to 34 Years | 2013 | <5 | 245 | 20.4 | ( 6.7,47.0) | 5900 | 59741 | 98.8 | ( 96.4,101.2) | 78.3 | (58.4,98.3) |
|  |  |  |  |  |  |  |  |  |  |  |  |
| 35 to 39 Years | 2005 | 6 | 394 | 15.2 | ( 5.6,32.8) | 3202 | 65129 | 49.2 | (47.5,50.9) | 33.9 | (20.5,47.4) |
| 35 to 39 Years | 2006 | 6 | 382 | 15.7 | ( 5.8,33.9) | 3336 | 64118 | 52 | (50.3,53.8) | 36.3 | (22.4,50.2) |
| 35 to 39 Years | 2007 | <5 | 369 | 5.4 | ( 0.7,19.4) | 3399 | 62994 | 54 | (52.2,55.8) | 48.5 | - |
| 35 to 39 Years | 2008 | <5 | 365 | 11 | ( 3.0,27.8) | 3384 | 61195 | 55.3 | (53.5,57.1) | 44.3 | - |
| 35 to 39 Years | 2009 | <5 | 352 | 5.7 | ( 0.7,20.4) | 3357 | 58869 | 57 | (55.2,58.9) | 51.3 | - |
| 35 to 39 Years | 2010 | <5 | 328 | 12.2 | ( 3.3,30.9) | 3282 | 56998 | 57.6 | (55.7,59.5) | 45.4 | - |
| 35 to 39 Years | 2011 | <5 | 307 | 16.3 | ( 5.3,37.6) | 3266 | 54933 | 59.5 | (57.5,61.5) | 43.2 | (27.2,59.1) |
| 35 to 39 Years | 2012 | <5 | 299 | 16.7 | ( 5.5,38.6) | 3219 | 52823 | 60.9 | (58.9,63.0) | 44.2 | (27.9,60.6) |
| 35 to 39 Years | 2013 | <5 | 291 | 10.3 | ( 2.1,29.8) | 3065 | 52108 | 58.8 | (56.8,60.9) | 48.5 | - |
|  |  |  |  |  |  |  |  |  |  |  |  |
| 40 to 44 Years | 2005 | 0 | 475 | 0 | (0.0,7.7) | 630 | 67589 | 9.3 | ( 8.6,10.1) | 9.3 | - |
| 40 to 44 Years | 2006 | <5 | 457 | 2.2 | ( 0.1,12.1) | 630 | 67765 | 9.3 | ( 8.6,10.0) | 7.1 | - |
| 40 to 44 Years | 2007 | <5 | 442 | 6.8 | ( 1.4,19.7) | 657 | 67574 | 9.7 | ( 9.0,10.5) | 2.9 | - |
| 40 to 44 Years | 2008 | <5 | 424 | 11.8 | ( 3.8,27.3) | 714 | 67152 | 10.6 | ( 9.9,11.4) | -1.2 | (-12.6, 10.3) |
| 40 to 44 Years | 2009 | <5 | 418 | 2.4 | ( 0.1,13.3) | 752 | 66339 | 11.3 | (10.5,12.2) | 8.9 | - |
| 40 to 44 Years | 2010 | <5 | 394 | 2.5 | ( 0.1,14.1) | 728 | 65355 | 11.1 | (10.3,12.0) | 8.6 | - |
| 40 to 44 Years | 2011 | <5 | 382 | 10.5 | ( 2.9,26.6) | 815 | 64715 | 12.6 | (11.7,13.5) | 2.1 | - |
| 40 to 44 Years | 2012 | <5 | 369 | 8.1 | ( 1.7,23.6) | 771 | 63359 | 12.2 | (11.3,13.1) | 4 | - |
| 40 to 44 Years | 2013 | 0 | 365 | 0 | ( 0.0,10.1) | 709 | 61375 | 11.6 | (10.7,12.4) | 11.6 | - |

Note: Where individual cell counts were <5 we have summarized the information to maintain individual anonymity.

Supplementary Figure 1


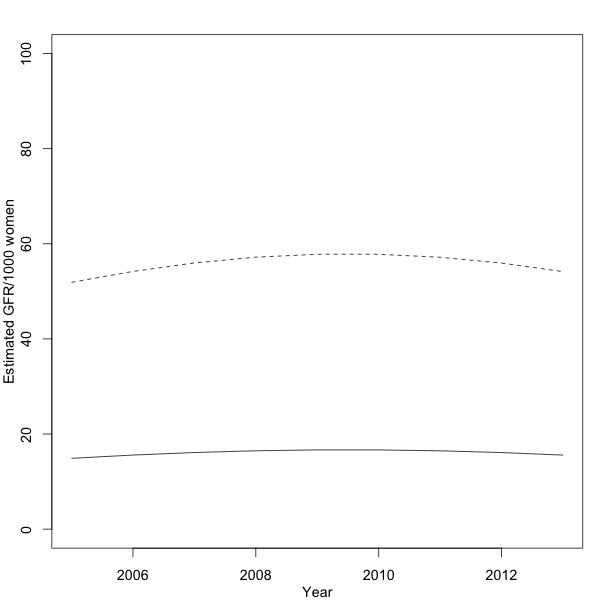


Supplementary Figure 2


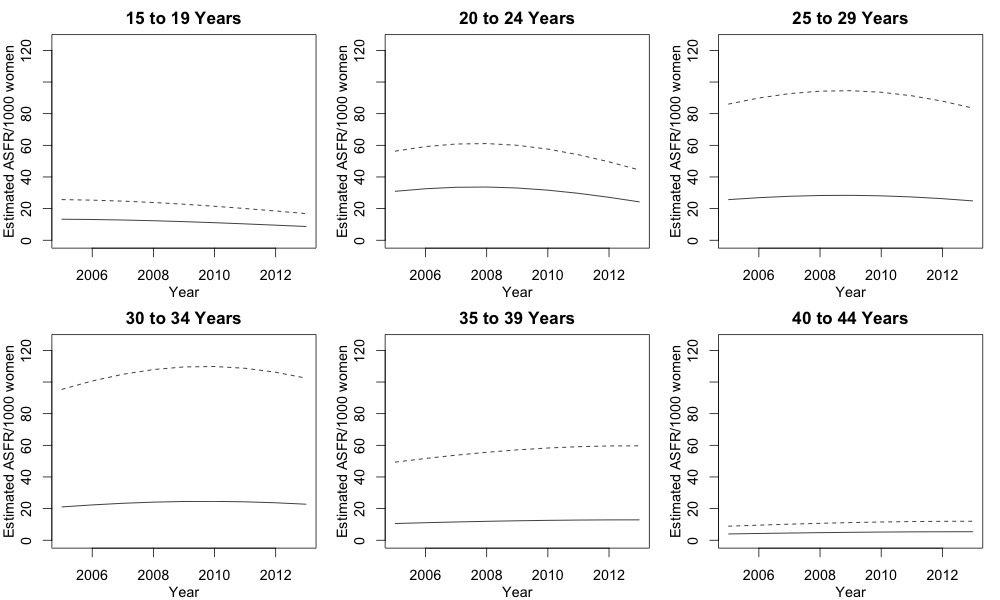

Supplement: Supplementary file 1 — Supplementary Table 1 Age-specific fertility rates (ASFR) per 1000 women amongst those aged 15–44 with a diagnosis of non-affective psychosis (exposed), compared with rates amongst women in the general population (unexposed) from 2005 to 2013. Supplementary Fig. 1 Estimated general fertility rates (GFR) per 1000 women for the population of exposed (solid line) and unexposed (dashed line) women between 2005 and 2013 Supplementary Fig. 2 Estimated age-specific fertility rates (ASFR) per 1000 women for the population of exposed (solid line) and unexposed (dashed line) women between 2005 and 2013 (DOCX 146 KB) [file 127_2022_2313_MOESM1_ESM.docx]
